# Increased Interleukin-17 in Peripheral Blood and Cerebrospinal Fluid of Neurosyphilis Patients

**DOI:** 10.1371/journal.pntd.0003004

**Published:** 2014-07-31

**Authors:** Cuini Wang, Lin Zhu, Zixiao Gao, Zhifang Guan, Haikong Lu, Mei Shi, Ying Gao, Huanbin Xu, X. Frank Yang, Pingyu Zhou

**Affiliations:** 1 STD Institute, Shanghai Skin Disease Hospital, Shanghai, People′s Republic of China; 2 Shanghai Skin Disease Hospital, Clinical School of Anhui Medical University, Shanghai, People′s Republic of China; 3 Department of Microbiology and Immunology, Indiana University School of Medicine, Indianapolis, Indiana, United States of America; University of Washington, United States of America

## Abstract

**Background:**

*Treponema pallidum* infection evokes vigorous immune responses, resulting in tissue damage. Several studies have demonstrated that IL-17 may be involved in the pathogenesis of syphilis. However, the role of Th17 response in neurosyphilis remains unclear.

**Methodology/Principal Findings:**

In this study, Th17 in peripheral blood from 103 neurosyphilis patients, 69 syphilis patients without neurological involvement, and 70 healthy donors were analyzed by flow cytometry. The level of IL-17 in cerebrospinal fluid (CSF) was quantified by ELISA. One-year follow up for 44 neurosyphilis patients was further monitored to investigate the role of Th17/IL-17 in neurosyphilis. We found that the frequency of Th17 cells was significantly increased in peripheral blood of patients with neurosyphilis, in comparison to healthy donors. IL-17 in CSF were detected from 55.3% neurosyphilis patients (in average of 2.29 (0–59.83) pg/ml), especially in those with symptomatic neurosyphilis (61.9%). CSF IL-17 was predominantly derived from Th17 cells in neurosyphilis patients. Levels of IL-17 in CSF of neurosyphilis patients were positively associated with total CSF protein levels and CSF VDRL (Venereal Disease Research Laboratory) titers. Notably, neurosyphilis patients with undetectable CSF IL-17 were more likely to confer to CSF VDRL negative after treatment.

**Conclusions:**

These findings indicate that Th17 response may be involved in central nervous system damage and associated with clinical symptoms in neurosyphilis patients. Th17/IL-17 may be used as an alternative surrogate marker for assessing the efficacy of clinical treatment of neurosyphilis patients.

## Author Summary

Syphilis, caused by the bacterium *Treponema pallidum*, can progress to affect the central nervous system (CNS) and cause damage in the brain and spinal cord, which is called neurosyphilis. While many neurosyphilis patients may not have any symptoms, some patients develop severe symptoms which can be life-threatening. Th17 cells are a subset of CD4^+^ T helper cells producing the hallmark cytokine IL-17, which are essential for effective antimicrobial host defense and are also involved in tissue damage. In this study we conduct a comparative analysis of Th17/IL-17 in the peripheral blood and cerebrospinal fluid (CSF) of syphilis patients without neurological abnormalities, and neurosyphilis patients with or without symptoms. Th17 frequency in peripheral blood was significantly increased in neurosyphilis. CSF IL-17 was increased in neurosyphilis patients, especially in those with symptomatic neurosyphilis. Levels of CSF IL-17 in neurosyphilis patients were positively associated with CNS damage. Notably, neurosyphilis patients with undetectable CSF IL-17 had better outcome upon treatment. These findings indicate that the Th17 response may be involved in central nervous system damage, clinical symptoms and prognosis of treatment of neurosyphilis patients.

## Introduction

Syphilis, a sexually transmitted multi-stage disease caused by the spirochete *Treponema pallidum*, remains to be a global public health problem with an estimated 12 million new cases annually [1]. In recent years, China has experienced a resurgence of syphilis cases, with the national incidence rate of 32.04 per 100,000 population and with 429,677 new cases reported in 2011 [2]. *T. pallidum* invades the human host through genital or oral mucosa, abraded skin, enters lymphatic system and bloodstream, and then disseminates to different organs. Without treatment, this spirochetal pathogen is able to survive in the human host for several decades, causing damage in multiple organs including nervous system (neurosyphilis) [3], [4].

Neurosyphilis is a frequent and protean clinical manifestations ranging from headache and oculopathy to more serious conditions such as cerebrovascular events, paretic and tabes dorsalis [5]. The mechanisms underlying the development of neurosyphilis remain poorly understood. *T. pallidum* can invade the CNS at any stage of infection and provokes robust cellular immune response [6]. In the non-human primate models, strong T helper (Th) 1-type immune response can contribute to the clearance of *T. pallidum* in CNS [6]. The immune response elicited during infection, although aimed to eliminate organisms, may also contribute to the pathogenesis. Cytokines produced by T lymphocytes are critical for regulation of both protective and pathogenic immune responses [7].

Th17 cells, with the hallmark of producing cytokine IL-17, were identified as a subset of CD4^+^ T helper cells. Emerging evidence has demonstrated that Th17 cells contribute to clearance of diverse organisms (*Mycobacterium tuberculosis*, *Pneumocystis carinii*, *Candida albicans* and *Klebsiella pneumonia et al.*) [8], [9], [10], [11]. On the other hand, Th17 also mediates strong immunopathology in chronic infection. Anti-IL-17 and anti-IL-17R treatments could prevent severe *Borrelia*-induced destructive arthritis [12]. Hence, Th17 response in infection may be involved in both protection and progression/chronic infection.

Previous studies reported an increase of IL-17 in secondary syphilitic lesion and peripheral blood [13], [14]. Recently, *Pastuszczak et al.* also showed elevated CSF IL-17 levels in early asymptomatic neurosyphilis [15], suggesting that IL-17 may be involved in local immune response to *T. pallidum* infection. In this study, we performed a comparative analysis of Th17/IL-17 in peripheral blood and CSF in asymptomatic and symptomatic neurosyphilis patients, and evaluated the relationship between CSF IL-17 level and the clinical outcomes. Our results suggested that Th17/IL-17 is a contributing factor to the immunopathology of neurosyphilis, and may be used to monitor the prognosis of treatments of syphilis infected patients.

## Methods

### Ethics statement and subjects

This study was performed at the Shanghai Skin Disease Hospital between Aug. 2010 and Dec. 2012. The study was approved by the Ethics Committee of the Shanghai Skin Disease Hospital. Written informed consents were obtained from all participants. Patients were identified and referred for enrollment by dermatologists, neurologists, psychiatrists and ophthalmologists after careful examination and evaluation. Syphilis was diagnosed at each stage of infection by a combination of compatible history, clinical features and the results of nontreponemal and treponemal tests of serum and CSF samples. The exclusion criteria include positive HIV infection; prior history of syphilis infection, or history of syphilis treatment (except for 7 serofast patients); history of systemic inflammation, autoimmune disease, other underlying acute or chronic disease, receiving anti-inflammatory medications, immunocompromised conditions, or use of antibiotics or immunosuppressive medications in the last four weeks. 70 healthy donors, who visited Shanghai Skin Disease Hospital voluntarily for a medical check-up for the purpose of STD prevention, were recruited to the study. All healthy subjects were negative for HIV and serological tests for syphilis (i.e., both serum RPR and TPPA negative), and did not have any clinical symptoms consistent with *T. pallidum* infection.

In this study, three groups of patients were included: 1) neurosyphilis group (including 40 subjects with asymptomatic neurosyphilis, 4 with meningovasculitis, 39 with general paresis, 8 with tabes dorsalis, and 12 with ocular neurosyphilis); 2) non-neurosyphilis group with normal CSF WBC count, CSF protein concentration and CSF-VDRL negative (including 13 subjects with primary syphilis, 30 with secondary syphilis, 19 with latent syphilis, and 7 with serofast syphilis); 3) 70 healthy donors. In this study, the serofast state must be met the following three criteria: i) syphilitic patients, despite receiving recommended standard treatment (according to Chinese National STI Treatment Guidelines), whose nontreponemal test titers (RPR) persists positive for at least two years of follow-up evaluation. ii) patients who denied high risk sexual behaviors (re-infection) following treatments; and iii) patients with RPR titers declined fourfold within 6 months after therapy. Peripheral blood from healthy donors was used for peripheral blood mononuclear cells (PBMC) isolation and for measurement of the baseline of the levels of IL-17^+^ cells and the frequency of Th17 cells. Since it is difficult to collect CSF from healthy donors, we used a separate control group of 29 patients who underwent orthopaedic or stone surgery (gall stone, kidney stone) but were serum RPR and TPPA negative, whose CSF samples were collected prior to spinal anaesthesia. The baseline level of IL-17 in CSF was determined using samples from the control group. All groups were well matched in the categories of gender and age. Additional information on the patient groups were presented in **Table 1**. CSF samples were stored at −70°C and thawed once before analyses.

**Table 1:** Clinical and laboratory characteristics of the control group and the syphilis patients.

	Healthy donors	Control	Non-neurosyphilis^a^	Neurosyphilis
**No. of Cases**	70	29	69	103
**Male No. (%)**	48(68.5)	18(62.1)	43(62.3)	73 (70.9)
**Age (range), y**	44(20–60)	46(18–65)	43(16–77)	50(25–75)
b **Duration (range), mo**	—	—	1(0.2–168)	8(0.1–72)
**Baseline plasma RPR (range)**	0(0–0)	0(0–0)	64 (1–512)	64 (1–512)
**CSF protein, mg/dl(range)**	ND	23(10–40)	26(10–44)	45 (14–186)
**CSF-WBC, cells/µl (range)**	ND	1(0–5)	1(0–5.5)	5.5(0–145.2)
**CSF VDRL (+) (%)**	ND	0(0)	0 (0)	99 (96.1)
**CSF VDRL(−) and CSF-TPPA(+) (%)**	ND	0(0)	10 (14.5)	4 (3.9)

Data are given as median (range), or frequencies.

a include 13 subjects with primary syphilis, 30 subjects with secondary syphilis, 7 subjects with serofast syphilis and 19 subjects with latent syphilis.

b “Duration of primary, secondary syphilis and symptomatic neurosyphilis” means the duration of clinical manifestations; “Duration of latent syphilis” means the period between the patient's high risk behaviors which may be infected with *T. pallidum* and the confirmed both serum TPPA/RPR positive. “Duration of asymptomatic neurosyphilis” means the period between the patient's high risk behaviors which may be infected with *T. pallidum* and the confirmed neurosyphilis. “Duration of the serofast” means the period between syphilitic patients who receiving recommended standard treatment, whose nontreponemal test titers remain positive and then was diagnosed as serofast.

**Abbreviations:** RPR, rapid plasma regain; VDRL, venereal disease research laboratory; TPPA, treponema pallidum particle agglutination assay; WBC, white blood cells; ND, not done.

**Healthy donors**: peripheral blood mononuclear cells (PBMC) isolation from healthy donors and for measurement of the baseline of the levels of IL-17^+^ cells and the frequency of Th17 cells. **Control:** patients who undergone orthopaedic or stone surgery and had not experienced syphilis, where CSF was collected prior to spinal anaesthesia. Samples from this group were used for measuring the baseline of the levels of IL-17 in CSF.

### Diagnostic criteria for neurosyphilis

First, all neurosyphilis patients have positive serum RPR and TPPA tests. The diagnosis of *confirmed neurosyphilis* includes reactive CSF-VDRL (Venereal Disease Research Laboratory) and CSF-TPPA tests in the absence of substantial contamination of CSF with blood. *Presumptive neurosyphilis* was defined as a nonreactive CSF-VDRL test but reactive CSF-TPPA with either or both of the following: (i) CSF protein concentration >45 mg/dl or CSF white blood cell (WBC) counts≥8/µl in the absence of other known causes for the abnormalities; (ii) clinical neurological or psychiatric manifestations consistent with neurosyphilis without other known causes for such abnormalities [16], [17].

Neurosyphilis is categorized as: asymptomatic, meningovascular, paretic, ocular and tabetic neurosyphilis. *Asymptomatic neurosyphilis* is defined by the presence of CSF abnormalities consistent with neurosyphilis and the absence of neurological/psychiatrical symptoms or signs. *Meningovasculitis* is defined by clinical features of meningitis and magnetic resonance image (MRI) evidence of brain lesions and/or a stroke syndrome. *General paresis* is characterized by personality changes, dementia and psychiatric symptoms including mania or psychosis. Patients with sensory loss, ataxia, lancinating pains, bowel and bladder dysfunction were considered as *Tabes dorsalis*. *Ocular neurosyphilis* (those who had ocular signs or symptoms but with normal CSF index were not included in this study)is defined by the presence of CSF abnormalities consistent with neurosyphilis and ocular signs or symptoms (worsening visual acuity and visual fields, floaters, papillitis, uveitis). All these forms of neurosyphilis should have no other known causes for these clinical abnormalities. A complete list of information of neurosyphilis patients were listed in **Table 2**.

**Table 2:** Clinical and laboratory features of the neurosyphilis patients enrolled in this study.

	Asymptomatic^b^	Symptomatic			
		Meningovascular	Paretic	Tabetic	Ocular
**No. of Cases**	40	4	39	8	12
**Male No. (%)**	23 (57.5)	4 (100)	35 (89.7)	5(62.5)	6(50)
**Age (range), y**	51.5(25–75)	47.5(41–54)	50(37–71)	48(34–59)	50(39–66)
a **Duration (range), mo**	2(0.1–12)	8(6–12)	12(0.5–60)	12(2–72)	12(6–36)
**Baseline plasma RPR(range)**	64(1–512)	48(2–128)	64(8–512)	32(2–128)	96(16–256)
**CSF protein, mg/dl(range)**	39.6(20–87)	50.5(22.3–100.5)	59.5(20–186)	33.9(14–64)	39.5(20–74)
**CSF-WBC, cells/µl (range)**	4.2(0–84)	40.7(5.6–73.7)	5(0–97.9)	19.8(2–145.2)	2.75(1–86.9)
**CSF VDRL (+) (%)**	40(100)	4(100)	37(94.9)	8(100)	10(83.3)
**CSF VDRL (−) and CSF-TPPA (+) (%)**	0(0)	0(0)	2(5.1)	0(0)	2(16.7)

Data are given as median (range), or frequencies.

a “Duration of asymptomatic neurosyphilis” means the period between the patient's high risk behaviors which may be infected with *T. pallidum* and the confirmed neurosyphilis. “Duration of symptomatic neurosyphilis” means the duration of clinical manifestations;

b includes 16 subjects with secondary syphilis, 2 subjects with early latent and 22 subjects with latent or unknown duration.

### The follow-up protocol

According to Chinese National STI treatment Guidelines, syphilis patients without CNS involvement were treated with benzathine penicillin 2.4MU/qw intramuscular for 1 or 2 weeks for early syphilis, and 3 weeks for late or unknown duration syphilis. If allergic to penicillin, ceftriaxone 0.25 g/day intramuscular for 10 days were given. Neurosyphilis patients were given aqueous crystalline pencillin G, 4MU intravenously every 4 h for 14 days, or ceftriaxone intravenously with 2 g daily for 10 days if allergic to penicillin. In the 103 neurosyphilis patients, 80 patients were treated with aqueous crystalline pencillin G, 4MU intravenously every 4 h for 14 days, 23 patients were treated with ceftriaxone intravenously with 2 g daily for 10 days.

All patients were asked for follow-up after treatment. Patients were selected if the patient's written informed consent was obtained. The exclusion criteria include positive HIV infection; history of syphilis or syphilis treatment; history of systemic inflammatory, autoimmune disease, other underlying acute or chronic disease, patients receiving anti-inflammatory medications, immunocompromised, or using antibiotics or immunosuppressive medications in the last four weeks.

In this study, 44 neurosyphilis patients were enrolled and followed up at Shanghai Skin Disease Hospital. Patients returned for follow-up visits at 3, 6, 9 and 12 months after treatment. All patients underwent lumbar puncture at the 3-month visit, and lumbar punctures were repeated at 6, 9 and 12 months if the previous CSF profile was abnormal. Blood samples were collected at each follow-up visits. All patients completed their 12 months follow-up visit.

For the CSF-VDRL and the serum RPR test, a 4-fold decrease or more in titer or reversion to a nonreactive result was defined as a normal response. Stepwise Cox regression models were used to determine the influence of the following factors on the likelihood of normalization of each measure and the improvement of clinical symptoms: (1) neurosyphilis treatment regimen (intravenous ceftriaxone, vs. intravenous aqueous penicillin G); (2) syphilis stage (secondary and early latent vs. late latent and syphilis of unknown duration); (3) baseline laboratory values (greater or less than the median value for those subjects with each abnormality); (4) CSF IL-17 levels: CSF IL-17 negative (<0.5 pg/ml) and CSF IL-17 positive (≥0.5 pg/ml); and (6) clinical symptoms.

### Preparation and stimulation of PBMC and CSF cells

Whole blood samples (5 ml) from syphilis patients and healthy donors were used for peripheral blood mononuclear cells (PBMC) isolation. PBMC were purified from peripheral blood by centrifugation using a Ficoll-Hypaque gradient (Axis-Shield). Because resting cells do not normally produce cytokines, cells were stimulated *in vitro* in order for the respective cytokine genes to be activated for intracellular cytokine staining. Phorbol myristate acetate (PMA) and ionomycin are unspecific stimulator that trigger a strong production of cytokines *in vitro* and are widely used to evaluate intracellular cytokine production from various T lymphocyte subpopulations [18]. Monensin is used to prevent the intracellular transport of cytokines from Golgi apparatus for enhancing the sensitivity of the detection. Accordingly, PBMC were seeded into 24-well culture plates (Corning) at 2×10^6^ cells/well and stimulated ex vivo with PMA (50 ng/ml) (Sigma) and ionomycin (1 µg/ml) (Sigma) for 4 hours. Monensin (2 uM) (eBioscience) was then added at the start of stimulation. CSF (10 ml) was centrifuged at 4°C immediately after spinal tap, and cells were stimulated as described above.

### Flow cytometric analysis

For intracellular staining, cells were first stained with ECD-labeled anti-human CD3 (Clone UCHT1, Beckman), FITC-labeled anti-human CD4 (Clone RPA-T4, Biolegend) and then fixed and permeabilized using Perm/Fix solution (Biolegend) at room temperature for 20 minutes. Cells were washed with Perm/Wash buffer (Biolegend) and stained with PE-labeled anti-human IL-17A (Clone BL168, Biolegend). Mouse IgG1 and IgG2 (BD Biosciences) were used as isotype controls. After staining, cells were analyzed with Epics XL (Beckman Coulter) and FlowJo software (Tree Star). Lymphocytes were gated according to forward and side scatter characteristics and CD4^+^T cells were gated based on CD3 and CD4 expression. IL-17 positive lymphocytes, CD3^+^, CD4^+^ T cells were defined by setting regions with the lower limits for cytokine positivity determined from isotype antibody.

### Measurement of IL-17 levels in CSF

IL-17 levels in CSF were determined using human IL-17 Quantikine ELISA kits (eBioscience) according to manufacturer's instruction. The sensitivity for detecting IL-17 is 0.5 pg/ml.

### Statistical analysis

Data were presented as median and range (min, max). Differences between the groups were analyzed using the nonparametric Mann-Whitney U test. The detection rates between the groups were assessed using χ^2^ test or Fisher's exact test. Spearman correlation analysis was performed between the levels of IL-17 and other parameters. Stepwise Cox regression models were used to determine the influence of the factors on the likelihood of normalization of each measure. All statistical analyses were performed using SPSS 17.0 software. A value of P<0.05 was considered significant.

## Results

### Increase of Th17 cells in the peripheral blood of neurosyphilis patients

**Figure 1:**
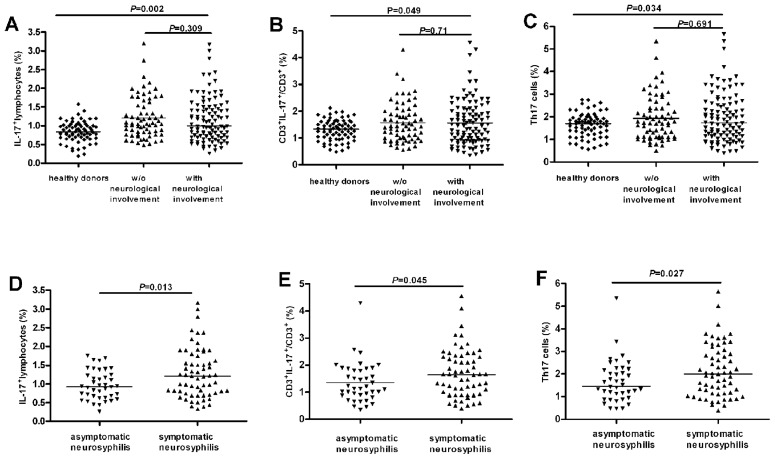
Increased IL-17^+^ and Th17 cells in peripheral bloods of neurosyphilis patients. Peripheral blood mononuclear cells (PBMC) were stimulated and stained for flow cytometric analysis. Lymphocytes were gated according to forward and side scatter characteristics, and CD4^+^ T cells were gated based on CD3 and CD4 expression. (A) The percentage of total IL-17^+^ cells in gated lymphocytes in peripheral bloods of healthy donors (n = 70), syphilis patients without neurological disorders (including primary, secondary, latent and serofast syphilis; n = 69), and syphilis patients with neurosyphilis (n = 103) (including both asymptomatic and symptomatic neurosyphilis). (B) The percentage of Th17 cells in gated CD3^+^ T cells in peripheral bloods of the three groups shown in (A). (C) The percentage of Th17 cells in gated CD4^+^ T cells in peripheral bloods of the three groups shown in (A). (D) The percentage of total IL-17^+^ lymphocytes in gated lymphocytes in peripheral bloods of asymptomatic (n = 40) and symptomatic (including meningovascular, paretic, ocular and tabetic, n = 63) neurosyphilis patients. (E) The percentage of Th17 cells in gated CD3^+^ T cells in peripheral bloods of the two groups as in (D). (F) The percentage of Th17 cells in gated CD4^+^ T cells in peripheral bloods of the two groups as in (D). Each dots represents one individual. Results represent the median + individual values.

To investigate the potential role of Th17 in neurosyphilis, we first examined the frequency of IL-17^+^ among lymphocytes, CD3^+^, CD4^+^ T populations in PBMC. The baseline frequency of total IL-17^+^ cells, and IL-17^+^ CD3^+^cells, and IL-17^+^ CD4^+^ T cells (Th17) of PBMC in healthy individuals were 0.86% (0.19%–1.58%), 1.33% (0.48%–3.2%) and 1.7% (0.56%–2.76%), respectively (**Fig. 1A, 1B & 1C**). We observed a significant increase in the frequencies of IL17^+^, CD3^+^IL-17^+^ and Th17 cells in syphilis patients with either non-neurosyphilis or neurosyphilis compared to those in healthy individuals (**Fig. 1A, 1B & 1C**). However, there was no significant difference in the frequencies of IL-17^+^ cells, CD3^+^IL-17^+^ and Th17 cells in PBMC between syphilis patients without neurological involvement (including primary, secondary, latent and serofast syphilis patients) and neurosyphilis patients (**Fig. 1A, 1B & 1C**).

To further investigate whether Th17 cells in peripheral blood are different between diverse clinical presentations of neurosyphilis, we divided neurosyphilis patients into two groups, asymptomatic (n = 40) and symptomatic neurosyphilis patients (n = 63). We then compared the Th17 cell frequency in PBMC between these two groups. As shown in **Fig. 1D, 1E & 1F**, patients with symptomatic neurosyphilis had significant higher percentage of total IL-17^+^cells, CD3^+^IL-17^+^ and Th17 in PBMC than the patient group with asymptomatic neurosyphilis.

### Increased IL-17 levels in cerebrospinal fluid (CSF) in neurosyphilis patients

**Figure 2:**
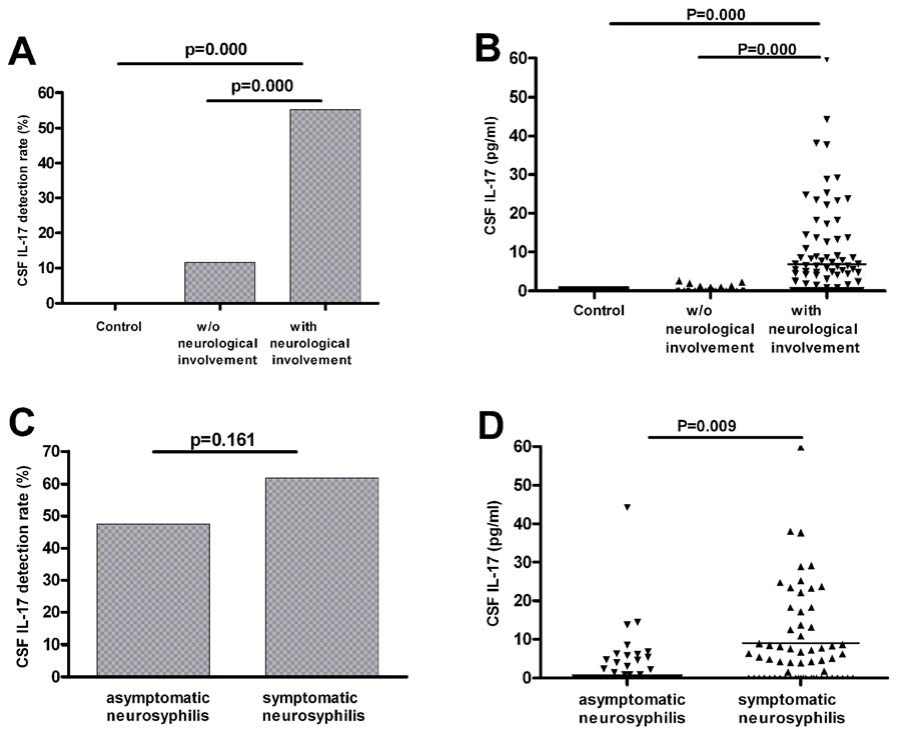
Increased CSF IL-17 levels in patients with neurosyphilis. (A) Detection rates of CSF IL-17(≥0.5 pg/ml) in control group (n = 29), syphilis patients without neurological disorders (including primary, secondary, latent and serofast syphilis; n = 69), or syphilis patients with neurological involvement (including asymptomatic and symptomatic neurosyphilis; n = 103). (B) Levels of CSF IL-17 proteins among the samples shown in (A). (C) Detection rates of CSF IL-17 in patients with asymptomatic (n = 40) or symptomatic (including meningovascular, paretic, tabetic and ocular; n = 63) neurosyphilis. (D) Levels of CSF IL-17 proteins among the CSF samples shown in (C). Results represent the median + individual values.

Since neurosyphilis patients had increased levels of Th17 cells in peripheral blood, we further investigated the IL-17 levels in CSF of these patients. We first compared the detection rate of IL-17 in CSF between neurosyphilis patients and non-neurosyphilis patients. We found that there was five-fold higher detection rate of IL-17 in CSF in neurosyphilis patients than that in non-neurosyphilis patients (**Fig. 2A**). The average levels of CSF IL-17 was also significantly higher in neurosyphilis patients (2.29 pg/ml) (range of 0–59.83 pg/ml) than that in non-neurosyphilis patients (0 pg/ml) (range of 0–2.60 pg/ml) (**Fig. 2B**). IL-17 was not detected in CSF of the control group (**Fig. 2A & B**).

We further compared the levels of CSF IL-17 between patients with asymptomatic and symptomatic neurosyphilis. The detection rates of CSF IL-17 were 47.5% and 61.9% in asymptomatic and symptomatic neurosyphilis, respectively. The level of CSF IL-17 in symptomatic neurosyphilis patients (4.91 pg/ml, range from 0 to 59.83 pg/ml) was significantly higher than that in asymptomatic neurosyphilis patients (0.715 pg/ml, range from 0 to 44.27 pg/ml). Noted that the symptomatic neurosyphilis patient group included meningovascular, paretic, ocular and tabetic neurosyphilis. Further examination showed that the level of CSF IL-17 was the highest among paretic patients (7.6 pg/ml, range from 0 to 38.07 pg/ml) (**Table 3**).

**Table 3:** Levels of IL-17 in CSF from patients with different clinical states of syphilis.

	Detection rate of IL-17(%)	IL-17(pg/ml)		
			*p* a	*p* b
**Control**	0(0/29)	0	—	—
**Non-neurosyphilis**	11.6(8/69)	0(0–2.60)	—	—
**Asymptomatic**	47.5(19/40)	0.715(0–44.27)^a^	*p* = 0.000	—
**Meningovascular**	25(1/4)	0(0–8.93)	*p* = 0.340	*p* = 0.608
**Paretic**	76.9(30/39)	7.6(0–38.07)*^ab^*	*p* = 0.000	*p* = 0.000
**Tabetic**	50(4/8)	0.78(0–18.23)^a^	*p* = 0.002	*p* = 0.571
**Ocular**	50(6/12)	0.92(0–59.83)^a^	*p* = 0.000	*p* = 0.640

Data are given as median (range), or frequencies,

a v.s. syphilis patients without neurological involvement.

b v.s. asymptomatic neurosyphilis patients.

IL-17 levels represent the median (range).

### The CSF IL-17 level positively correlates with CSF protein concentration and CSF VDRL titer in neurosyphilis patients

**Figure 3:**
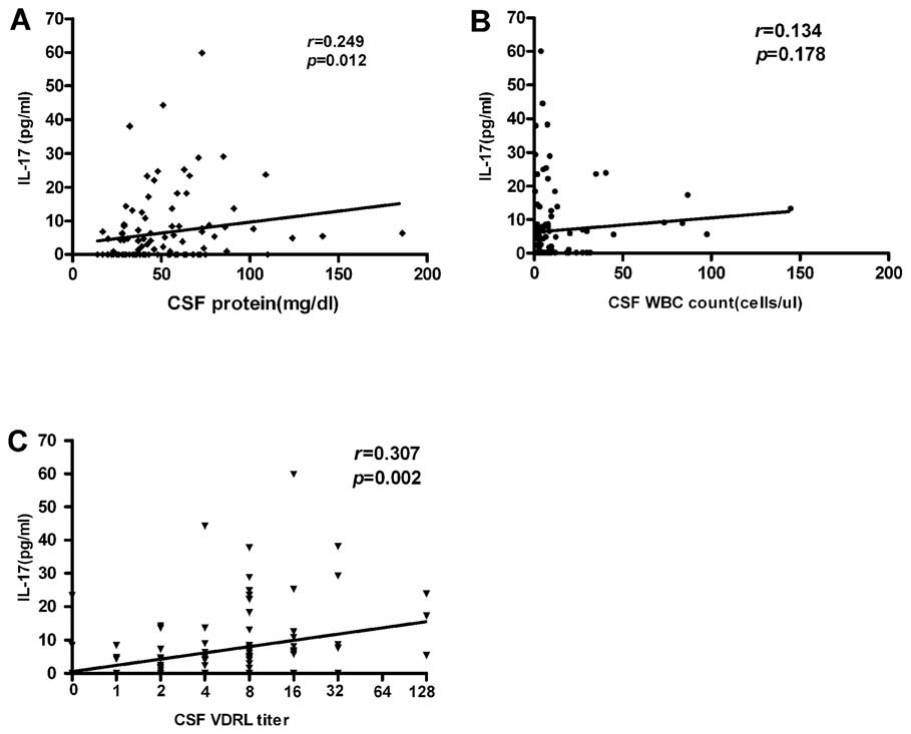
Correlations between the CSF IL-17 levels and the CSF protein levels, CSF WBC counts or CSF VDRL titer of neurosyphilis patients. CSF protein levels (A), CSF WBC counts (B) and CSF VDRL titer (C) are plotted against CSF IL-17 level in neurosyphilis patients (n = 103). Each dot represents an individual patient. The straight line in each graph is the result of linear regression analysis. Spearman's correlation coefficients (*r*) and *P* values are shown.

*T. pallidum* is capable of invading central nervous system and damaging local tissues. There are detectable CSF abnormalities in neurosyphilis patients, including positive CSF VDRL, pleocytosis, and/or elevated protein concentration [19]. These measurements correlate well with the disease activity [19]. Since the above data showed that neurosyphilis patients had increased CSF IL-17 levels, we further investigated a possible relationship between CSF IL-17 levels and other measurements. As shown in **Fig. 3**, there was a significant positive correlation between CSF IL-17 levels and CSF protein concentrations or CSF VDRL titer, but not with the CSF WBC counts.

In some neurosyphilis patients, CSF IL-17 was not detected. We thus further investigated whether there are certain factors which may contribute to this phenomenon. We found that there were no differences in age, sex, the baseline serum RPR titer, or duration of symptoms prior to diagnosis between the IL-17 positive and IL-17 negative neurosyphilis groups. However, the IL-17 positive group had higher CSF protein concentration and CSF VDRL titer and higher frequency of symptomatic neurosyphilis than that of the IL-17 negative group (**Table 4**).

**Table 4:** Comparison of clinical features between patients with CSF IL-17^+^ and IL-17^−^ neurosyphilis.

	IL-17 negative	IL-17 positive
**No. of Cases**	45	58
**Male No. (%)**	32(71.1)	45(77.6)
**Age (range), y**	52(25–72)	49(26–75)
a **Duration (range), mo**	10(0.1–72)	12(0.1–60)
**Baseline Serum RPR titer (range)**	64 (1–256)	64(8–512)
**CSF-WBC, cells/µl (range)**	5.25(0–31.9)	5.6(0–145.2)
**CSF protein, mg/dl(range)**	40(14–110)	51(17–186)*
**CSF VDRL titer (range)**	4(0–32)	8(0–128)**
**Symptomatic neurosyphilis**	48.9(22/45)	70.7(41/58)*

Data are given as median (range), or frequencies.

*significant differences between the two groups,*p<0.01.

**significant differences between the two groups, **p<0.001.

a “Duration of asymptomatic neurosyphilis” means the period between the patient's high risk behaviors which may be infected with *T. pallidum* and the confirmed neurosyphilis. “Duration of symptomatic neurosyphilis” means the duration of clinical manifestations.

### Th17 are dominant IL-17-producing cells in CSF of neurosyphilis patients

**Figure 4:**
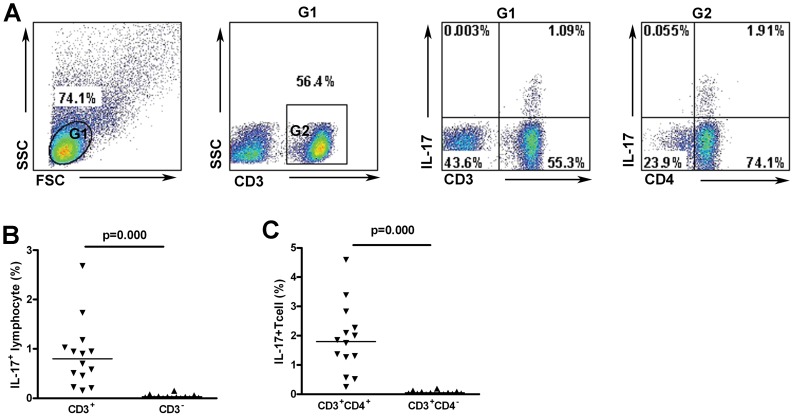
Th17 frequencies in CSF lymphocytes from neurosyphilis patients. (A) Representative dot plot of CSF IL-17-producing cells in CD3^+^CD4^+^, CD3^+^CD4^−^ and CD3^−^ lymphocytes from neurosyphilis patients. (B) Frequencies of IL-17^+^ cells in the populations of CD3^+^, CD3^−^ lymphocytes (n = 14). (C) Frequencies of IL-17^+^ cells in the populations of CD3^+^CD4^+^, CD3^+^CD4^−^ lymphocytes (n = 14). Results represent the median + individual values.

We further analyzed IL-17-producing cells in CSF of neurosyphilis patients. Because of the limited sample sizes and lymphocyte cell numbers in CSF collected from neurosyphilis patients, CSF cells from 14 neurosyphilis patients who had high levels of CSF pleocytosis (>50 cells/ul) were collected and stimulated for intracellular staining for the purpose of this study. 14 subjects included 6 (42.9%) subjects with asymptomatic neurosyphilis, 5 (35.7%) subjects with paretic, 2 (14.3%) subjects with ocular neurosyphilis, 1 (7.14%) subjects with meningovascular neurosyphilis. The average percentage of CD4^+^ T cells was 58.28% (51.65%–80.1%) of total lymphocytes, and Th17 (CD3^+^CD4^+^IL-17^+^ cells) was 1.8% (0.25%–4.6%) (**Fig. 4**). However, Th17 cells accounted for 88.8% (45.1%–100%) of total IL-17^+^ cells (**Fig. 4**), indicating that Th17 is the dominant IL-17-producing cells and may play an important role in neurosyphilis.

### Increased CSF IL-17 were associated with poor prognosis of clinical treatment of neurosyphilis

Among 44 subjects with confirmed neurosyphilis, 22(50%) subjects were asymptomatic neurosyphilis, 15 (34.1%) were paretic neurosyphilis, 5(11.4%) were ocular neurosyphilis, 2 (4.5%) were meningovascular neurosyphilis. All enrolled patients were routinely under followed-up examination and treated with standard therapy according to the Chinese treatment Guidelines.

Factors that were included in the final regression models of normalization of each laboratory measure, the improvement of clinical symptoms and their HRs are shown in **Table 5**. Factors that may improve laboratory markers or clinical symptoms were analyzed using the final regression model (**Table 5**). The neurosyphilis treatment regimen did not influence normalization of any of the 4 laboratory markers and the improvement of clinical symptoms. Normalization of the CSF protein concentration was more likely to occur in subjects with early syphilis (p = 0.018). CSF-VDRL reactivity was less likely to become normal in patients with positive CSF IL-17 (p = 0.04) and with higher baseline CSF-VDRL titer (p = 0.019). Serum RPR reactivity was more likely to return to normal in subjects with higher baseline serum RPR titers (p = 0.008).

**Table 5:** Factors that influence normalization of each laboratory measure in the final Cox regression models.

Laboratory markers	HR(95%CI)				
	Stage of syphilis^a^	Treatment regimen^b^	Baseline value^c^	CSF IL-17 level^d^	clinical symptoms
**CSF-WBC**	NS	NS	NS	NS	NS
**CSF protein concentration**	6.62(1.38–31.68)	NS	NS	NS	NS
**CSF-VDRL reactivity**	NS	NS	0.40(0.18–0.86)	0.41(0.18–0.93)	NS
**Serum RPR test reactivity**	NS	NS	2.82(1.3–6.09)	NS	NS
**the improvement of symptoms**	NS	NS	NS	NS	NS

HR, hazard ratio; NS, in the stepwise model and therefore not included in the final model;

a Early (secondary and early latent) stage versus late latent stage and unknown duration;

b aqueous crystalline pencillin G,4MU intravenous every 4 h for 14 days versus ceftriaxone, 2 g intravenous daily for 10 days;

c Greater than the median value versus less than or equal to the median value in subjects with each abnormality. Median values were as follows: CSF WBC count, 8.8 cells/ml; CSF protein concentration, 68 mg/dl; CSF-VDRL titer, 1∶4; serum RPR test titer, 1∶64.

d CSF IL-17 positive (≥0.5 pg/ml) versus CSF IL-17 negative (<0.5 pg/ml).

## Discussion

*T. pallidum* remains one of the human pathogens that cannot be cultivated *in vitro* to-date. A suitable animal model for studying the pathogenesis of syphilis is also lacking. These obstacles have greatly hindered the effort of elucidating the basic immunobiological traits of syphilis. As a consequence, little is known about how *T. pallidum* causes damage to the central nervous system.

IL-17, a potent proinflammatory cytokine, plays a key role in the induction and development of tissue injury. IL-17 results in an increased production of ICAM-1, IL-6 and IL-8, and an increased synergy of many effects of IL-1β and TNF-α, which enhances the local inflammation and leads to inflammatory destruction [20], [21]. In this study, we observed an elevated CSF IL-17 in neurosyphilis patients. A similar scenario has been observed in infectious and auto-immune CNS disorders [22], [23]. Furthermore, we found that the level of CSF IL-17 is positively associated with CSF VDRL titer and total CSF protein in neurosyphilis patients. These findings suggest that IL-17 may involve in the CNS damage in neurosyphilis patients.

Syphilis is known as a “great imitator” because it is protean, especially neurosyphilis. Based on the patient's clinical and laboratory features, neurosyphilis is divided into five diagnostic categories, including asymptomatic, meningitis, meningovascular, paretic, and tabetic neurosyphilis [5]. Meningitis involves diffuse inflammation of the meninges with signs and symptoms of meningitis including headache, photophobia, nausea, vomiting, cranial nerve palsies, and occasionally seizures. It is diagnosed within 12 months after *T. pallidum* infection but it is relatively rare [5]. Unfortunately, no meningitis neurosyphilis patients enrolled in this study, and thus, the involvement of IL-17 in this stage of neurosyphilis remains unknown.

The pathogenic mechanisms underlying different clinical presentations of neurosyphilis are largely unknown. In this study, we found that higher levels of IL-17 were observed in CSF of symptomatic neurosyphilis patients, especially in paretic patients, compared with asymptomatic neurosyphilis patients. Moreover, the higher CSF protein and VDRL titer were observed in symptomatic neurosyphilis patients. These results suggest that IL-17 may be associated with clinical symptoms in neurosyphilis patients. Asymptomatic neurosyphilis does occur in both early and latent stages of syphilis. It is reported that there was an elevated CSF IL-17 level in early asymptomatic neurosyphilis patients, which correlated with the extent of CSF abnormality [15]. In our study, CSF IL-17 could be detected in 66.7% (12/18) of early asymptomatic neurosyphilis patients. It is believed that asymptomatic neurosyphilis patients may develop to late neurological complications [24]. Moreover, the extent of abnormalities of CSF positively correlated with the probability of developing late neurological complications [25]. Based on these notions, we suggested that some early asymptomatic neurosyphilis patients might have persistent IL-17 inflammation response, which could damage the CNS, resulting in neurological symptoms. Regrettably, there has been no study to compare long-term outcomes between CSF IL-17 negative and positive asymptomatic neurosyphilis patients.

Pastuszczak et al. identified that there was a strong correlation between CSF IL-17 and CSF pleocytosis in early asymptomatic neurosyphilis patients [15]. But our results indicated that there was no correlated between the level of CSF IL-17 and CSF pleocytosis. The CSF pleocytosis in neurosyphilis was related to the syphilis stage besides to the CNS damage. There was a marked pleocytosis in patients with acute meningeal syphilis. In late stage, CSF WBC counts in some neurosyphilis patients were less or even normal and were inconsistent with clinical symptoms. In up to 10% of patients with tabes referred to as the “burned out” stage, the CSF cell count may be normal [5]. In our study, there were early and late stage neurosyphilis patients. There were some paresis patients the CSF WBC counts were normal, though the clinical manifestations were severe. Therefore, according to the data, the CSF WBC counts were not always correlated with the degree of CNS damage. The different stage patients were enrolled in our study, leading to be inconsistent with the previous results.

Besides CD4^+^ T cells, other cells are capable of secreting IL-17 [26]. It was previously shown that IL-17 is mainly secreted by CD4^+^ T cells (Th17) in CSF in patients with chronic inflammatory demyelinating polyradiculoneuropathy (CIDP) [27]. In this study, we observed the CD4^+^ T cells were accumulated in CSF in neurosyphilis patients, and they were the dominant IL-17-producing cells. This finding suggests that Th17 response is a part of the local CNS response in a sub-population of neurosyphilis patients.

Our results showed that Th17 cells were increased in CSF of neurosyphilis patients. The mechanisms underlying the increase of Th17 in CNS remain unclear. IL-17 can disrupt the tight junction molecules and activates the endothelial contractile machinery, leading to disruption of blood brain barrier (BBB) [28]. Thus, Th17 in CSF may be a consequence of passive diffusion from blood. On the other hand, microbial lipopeptides such as *Helicobacter pylori* HP-NAP and *B. burgdorferi* NapA, can induce Th17 differentiation and production of IL-17 [29], [30]. In this regard, *T. pallidum* TpF1 is a protein homolog of HP-NAP and NapA [31], which may be capable of promoting Th17 differentiation and expansion in CNS. Interestingly, recent data showed that TpF1 can stimulate Treg cell differentiation [32]. The mechanisms underlying the increase of Th17 in CNS in neurosyphilis need to be further elucidated.

Because Th17 response may induce the immune-mediated CNS injury, we further evaluated the relationship between IL-17 and the clinical outcome of neurosyphilis. The baseline CSF-VDRL titer, and serum RPR titer influenced the likelihood of normalization of each parameter, consistent with previous studies [33]. However, we observed that CSF IL-17 positive neurosyphilis patients were 2.43 times less likely to normalize CSF-VDRL reactivity, even after taking into account the baseline CSF-VDRL titer and the stage of syphilis. CSF VDRL titer may reflect the *T. pallidum* burden and the extent of CNS damage. *T. pallidum* invaded CNS can induce Th17 immune response and CSF IL-17 were positively correlated with CSF VDRL titer. So positive CSF IL-17 in patients may reflect higher number of *T. pallidum* spirochetes in CSF. Since longer time would be required to clear a higher number of *T. pallidum* burden, the likelihood of normalization of CSF VDRL reactivity at the end of the observation would be lower. Because the sample size is limited (the number of subjects included in the regression analyses was only ranged from 21 to 44 patients in this study), a large sample study is needed to further understanding the true immune response at different stages of neurosyphilis and its clinical significance.

In conclusion, our findings demonstrate that neurological damage in syphilis patients is associated with increased CSF Th17/IL-17 response. CSF IL-17 may be used to evaluate the clinical outcome of treatment of neurosyphilis.
