# Correction: Increased Interleukin-17 in Peripheral Blood and Cerebrospinal Fluid of Neurosyphilis Patients

**DOI:** 10.1371/journal.pntd.0003842

**Published:** 2015-06-19

**Authors:** Cuini Wang, Lin Zhu, Zixiao Gao, Zhifang Guan, Haikong Lu, Mei Shi, Ying Gao, Huanbin Xu, X. Frank Yang, Pingyu Zhou

There is an error in the affiliation of the last author. Pingyu Zhou is also affiliated with Shanghai Skin Disease Hospital, Clinical School of Anhui Medical University, Shanghai, People′s Republic of China.
